# Quantitative Color Fundus Autofluorescence in Patients with Diabetes Mellitus

**DOI:** 10.3390/jcm10010048

**Published:** 2020-12-25

**Authors:** Stela Vujosevic, Caterina Toma, Paolo Nucci, Marco Brambilla, Stefano De Cillà

**Affiliations:** 1 Eye Clinic, IRCCS MultiMedica, 20123 Milan, Italy; 2 University Hospital Maggiore della Carità, Eye Clinic, 28100 Novara, Italy; tomacaterina@gmail.com (C.T.); stefano.decilla@med.uniupo.it (S.D.C.); 3 Department of Clinical Sciences and Community Health, University of Milan, 20123 Milan, Italy; paolo.nucci@unimi.it; 4 Department of Medical Physics, University Hospital Maggiore della Carità, 28100 Novara, Italy; marco.brambilla@maggioreosp.novara.it; 5 Department of Health Sciences, University East Piedmont “A. Avogadro”, 28100 Novara, Italy

**Keywords:** autofluorescence, green-emitting fluorophores, advanced glycation end products, lipofuscin, diabetes mellitus, diabetic retinopathy, diabetic macular edema, retinal inflammation

## Abstract

A new short wavelength confocal blue-light 450 nm-fundus autofluorescence (color-FAF) allows for visualization of minor fluorophores (e.g., advanced glycation end products, AGEs), besides lipofuscin. The aim of the present pilot study was to quantitatively evaluate color-FAF in patients with diabetes mellitus (DM) and to correlate these data with different stages of retinal disease severity. Optical coherence tomography and color-FAF images of 193 patients/eyes and 18 controls were analyzed using a custom software for quantification of the long (red) and short (green) wavelength components of the emission spectrum (REFC/GEFC). Measurements were performed in nine quadrants of the 6-mm ETDRS macular grid. Foveal GEFC and REFC intensities were higher in patients with DM compared to controls (*p* = 0.015 and *p* = 0.006 respectively) and in eyes with center involving diabetic macular edema (DME) compared to eyes without DME (*p* < 0.001). A positive correlation was found between GEFC and REFC intensities and central retinal thickness, *r* = 0.37 (*p* < 0.001) and *r* = 0.42 (*p* < 0.001), respectively. No differences were found in color-FAF among different DR severity groups. Quantitative color-FAF could become helpful for the metabolic evaluation of retina in patients with DM and in DME; however, further histologic and immunohistochemical studies on distribution of different retinal fluorophores in DM are needed to better understand its role.

## 1. Introduction

Diabetic retinopathy (DR) is a complex multifactorial disease in which the chronic increase in intracellular glucose determines the activation of four main metabolic pathways: the diacylglycerol-protein kinase; advanced glycation end products (AGE) and AGE receptors; sorbitol; and hexosamine pathways [1,2,3]. This aberrant metabolic response leads to cellular damage involving all retinal elements with consequent microvascular dysfunction, neuronal apoptosis, and abnormal inflammatory response with activation of glial cells [1,2,3,4].

Recently, the advent of optical coherence tomography (OCT) and OCT-angiography (OCT-A) have offered new insights into the non-invasive clinical evaluation of DR and diabetic macular edema (DME). Fundus autofluorescence (FAF) is another important non-invasive retinal imaging modality widely used for the evaluation of retinal pigment epithelium integrity in different chorio-retinal degenerations and dystrophies [5]. However, its use in patients with DR and DME is still limited, and its role not fully understood [6]. A direct correlation between the presence of increased FAF in the fovea and retinal volume and indirect correlation with retinal sensitivity was documented in DME when using conventional blue-light FAF at 488 nm [7], and a decrease of hyper-FAF after treatment with both intravitreal anti-vascular endothelial growth factor (VEGF) and steroids [8,9]. In addition, increased FAF in the fovea is considered an inflammatory biomarker of DME, as it is thought to be due to accumulation of oxidative products induced by activated microglial cells [7,10,11]. 

FAF intensity in the longer wavelength emission range (mostly the red spectrum, 580–700 nm) is mainly due to lipofuscin and, in the second instance, to N-retinylidene-N-retinylethanolamine (A2E). However, other minor fluorophores contribute to the final FAF intensity in the shorter wavelength emission range (green spectrum, 500–560 nm) [12]. Recently, a new short wavelength confocal blue-light FAF system with an excitation peak at 450 nm and which provides the so-called “color-FAF” has become commercially available (EIDON, CenterVue, Padua, Italy). This instrument allows for the detection of fluorescence due to minor fluorophores other than lipofuscin [12], such as AGEs, the oxidized fluorescent form flavin adenine dinucleotide (FAD) of the redox pair FAD-FADH2, and collagen 2 [13,14,15]. FAD (an intracellular molecule located in the mitochondria) and AGEs are considered important biomarkers of metabolic activity of the retina, while collagen plays a more structural role in the posterior structures of the eye [14,16,17,18,19]. 

The aim of the present pilot study was to quantitatively evaluate different emission components (short wavelength and long wavelength) of color-FAF in the macula of patients with diabetes mellitus (DM), with or without DR and DME; additionally, the study aimed to correlate quantitative data of color-FAF with systemic metabolic control, disease severity, and presence of DME.

## 2. Methods

### 2.1. Patients and Study Design

Three hundred and sixty-six patients with DM (with or without DR) were consecutively enrolled in this prospective, observational cross-sectional case evaluation. All patients included in the study were evaluated at the Medical Retina Service of the University Hospital Maggiore della Carità, Novara, Italy, between September 2018 and December 2019. Only one eye per patient, with a more severe stage of DR and DME (if present), was chosen for inclusion in the study; except better quality imaging in the other eye was present. In addition, 18 normal eyes of 18 healthy volunteers were recruited as the control group. 

Inclusion criteria for the present study were age ≥ 18 years; diagnosis of type 1 or type 2 DM according to the updated diagnostic criteria of the American Diabetes Association [20]; any stage of DR and diabetic maculopathy assessed by an expert ophthalmologist (S.V.) on slit-lamp fundus examination with a 90D lens (Volk Optical Inc., Mentor, OH, USA) according to the International Clinical Diabetic Retinopathy Disease Severity Scale [21], and confirmed by OCT for DME (central retinal thickness, CRT ≥ 300 μm) [22]; and pseudophakia or only mild cataract (not precluding good quality imaging of the retina). Exclusion criteria were any retinal disease other than DR and DME; presence of drusen or any other clinical sign of age-related macular degeneration (AMD); previous vitreoretinal surgery; use of any drug that could damage macular function (e.g., tamoxifen, hydroxychloroquine); or poor quality imaging. 

A full ocular and systemic medical history was recorded for each patient, in particular concerning DM: type and duration of DM, value of glycated hemoglobin (HbA1c), and use of antidiabetic agents (insulin and/or oral hypoglycemic drugs). All patients underwent a complete eye examination including best-corrected visual acuity (BCVA) at 4 meters using standard Early Treatment Diabetic Retinopathy Study (ETDRS) charts, dilated fundus examination, and non-invasive retinal imaging with color fundus photography (CFP), swept-source (SS) OCT, and color-FAF performed on the same day.

### 2.2. Imaging

#### 2.2.1. Swept-Source Optical Coherence Tomography

SS-OCT was performed using DRI SS-OCT Triton plus (Topcon Medical Systems Europe, Milano, Italy), and the acquisition protocol consisted of 6-mm radial OCT scans centered on the fovea. OCT scans were used to determine macular thickness (automatically provided by the instrument) in nine different quadrants of a standard 6-mm ETDRS grid and to detect the presence and site of intraretinal fluid (IRF; i.e., cysts).

#### 2.2.2. Color fundus photography and fundus autofluorescence

CFP and FAF were performed with a fully automated confocal light-emitting diode (LED) fundus imaging system (EIDON, CenterVue, Padua, Italy), acquiring 60 (horizontal) × 55 (vertical) degrees images (resolution 3680 × 3288 pixels) of the posterior pole with a single exposure. The blue-light FAF device has a peak excitation wavelength at 450 nm (range 440–475 nm) and emission detection between 500 and 750 nm. This device, equipped with a color sensor, provides so-called “color-FAF” images, consisting of only the red (range 560–700 nm) and green (range 500–560 nm) emission fluorescence components (REFC/GEFC). Color-FAF images were acquired and carefully checked by a trained grader (C.T.) to select only high quality images, since poor resolution (due for example to media opacity, eye-movements, or iris blockage) can significantly alter the results. The expert retinal specialist (S.V.) reviewed all questionable cases and gave the final adjudication on the quality of images.

#### 2.2.3. Color-FAF Images Analysis

**Figure 1:**
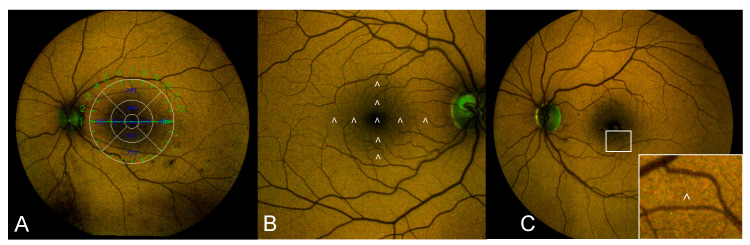
Example image showing the methodology used for color-FAF quantitative analysis. (**A**) Color-FAF of the posterior pole of a patient affected by DM and DR with superimposed standard ETDRS grid used as a reference for the identification of the 9 quadrants included in the analysis (corresponding values of retinal thickness are reported inside the grid); (**B**) Enlarged color-FAF of an healthy eye included as control showing the 9 sites where quantitative analysis of color-FAF parameters (GEFC/REFC intensity and emission wavelength) was performed (indicated by white arrowheads); (**C**) Color-FAF of a patient with moderate non-proliferative DR and DME showing in the white box at the bottom-right corner of the image a detail of the inner inferior quadrant: the site of analysis (indicated by the white arrowhead) was carefully chosen to select an area where no concomitant lesions (in this case microaneurysms and hemorrhages) were present. FAF: fundus autofluorescence; DM: diabetes mellitus; DR: diabetic retinopathy; ETDRS: early treatment diabetic retinopathy study; GEFC/REFC: green/red emission fluorescence components; DME: diabetic macular edema.

Color-FAF images were exported from the device and opened with the custom-made image analysis software “FAF Color Segmentation Tool” for quantitative evaluation. This specifically designed software separately measures the contribution of the long wavelength (red-R) and short wavelength (green-G) components of the emission spectrum (REFC and GEFC) for each pixel of the image (each pixel approximately corresponding to a square of 5 × 5 μm^2^). Hence, the software provides an estimation of the overall wavelength by means of an exponential model based on the proportion of GEFC and REFC. The software provides for each pixel of the image data on fluorescence intensity and emission wavelength displayed in a two-dimensional xy graph (segment graph), where emission wavelength is reported on the x-axis and intensity (calculated as (red + green)/2) on the y-axis. A second graph separately displays information on REFC and GEFC intensity on the two axes: the red component on the x-axis and the green component on the y-axis [23]. Values of emission wavelength and REFC and GEFC intensity were recorded in 9 different quadrants of the macular region using a standard 6-mm ETDRS grid as a reference. The sites chosen for the quantitative analysis were carefully selected to avoid the concomitant presence of retinal vessels or retinal lesions (e.g., microaneurysms, hemorrhages, hard exudates) that could alter the results (Figure 1).

### 2.3. Statistical Analyses

The means of the experimental groups were reported as arithmetic mean (standard deviation) for continuous variables in the text and Tables. Categorical variables were reported as experimental percentages.

Demographic variables were compared among different study groups using one-way ANOVA. The means of populations were estimated as least square means, which are the best linear estimates for the marginal means in the ANOVA design. In case of an overall statistically significant difference among study groups, pairwise comparisons among the different groups were done using Scheffé’s test.

A two-sided unpaired *t*-test was used for comparisons of clinical and color-FAF variables between patients with DM (only without IRF) and without DM (as well as for comparisons of color-FAF variables in different quadrants of the ETDRS grid between patients with or without DME. 

The color-FAF variables were compared among the patients with DM categorized according to the stage of retinopathy using a one-way ANOVA. The means of populations were estimated and reported in the tables as least square means, which are the best linear estimates for the marginal means in the ANOVA design. 

Correlation between color-FAF variables and metabolic control, duration of DM, and stage of DR was assessed by Pearson’s correlation coefficient as well as correlation between color-FAF variables and retinal thickness in different quadrants of the ETDRS grid. 

The statistical analyses were performed using Statistica software version 6.0 (StatSoft Inc., Tulsa, OK, USA), using a two-sided type I error rate of *p* ≤ 0 05.

## 3. Results

Of 366 patients (eyes) with DM and 18 eyes of 18 healthy controls initially enrolled in the study, 193 eyes and all 18 normal eyes were included in the final analysis. The reason for exclusion of 171 eyes was poor quality color-FAF imaging precluding reliable quantitative analyses. 

Twenty-five (13%) patients had type one DM and 168 (87%) patients type two DM. Thirty-nine (20.2%) eyes had no clinical signs of DR, 22 (11.4%) eyes had mild DR, 100 (51.8%) eyes had moderate DR, and 32 (16.6%) eyes had sight-threatening DR (20 eyes severe DR and 12 eyes proliferative DR). Forty-one eyes had center involving DME (with CRT ≥ 300 μm). The mean value of HbA1c was 6.7 ± 1.3% in patients with no DR, 7.2 ± 0.9% in patients with mild DR, 7.6 ± 1.3% in patients with moderate DR, and 7.4 ± 1.5% in patients with sight-threatening DR (*p* = 0.21). DM duration was significantly higher in patients with DR vs. patients with no DR (*p* < 0.001). The mean age was significantly lower in controls vs. patients with moderate DR (*p* = 0.03). Main clinical and demographic data (age, HbA1c, duration of DM, and BCVA) are summarized in Table 1.

Mean values of foveal GEFC and REFC intensity were significantly higher in patients with DM (with or without DR) compared to the control group (17.4 ± 14.1 vs. 9.1 ± 6.1 for GEFC, *p* = 0.015, and 18.5 ± 12.6 vs. 10 ± 6.6 for REFC, *p* = 0.006). Mean values of GEFC and REFC intensity in the 1 central mm of the ETDRS grid were significantly higher in eyes with DME compared to eyes without DME (43.3 ± 21.2 vs. 17.4 ± 14.1 as for GEFC, *p* < 0.001, and 47.1 ± 21.4 vs. 18.5 ± 12.6 as for REFC, *p* < 0.001). Mean values of GEFC and REFC intensity were significantly lower in eyes with IRF in the outer superior and outer temporal quadrants of the ETDRS grid compared to eyes without IRF in the same location (*p* = 0.03 and 0.004 as for GEFC and *p* = 0.001 and 0.002 as for REFC). Mean GEFC and REFC values in the other ETDRS quadrants were not significantly different between eyes with and without IRF and are reported in Table 2.

No significant correlations were found between color-FAF parameters and metabolic control (values of HbA1c), duration of DM, and DR severity (stage of DR) in patients with DM. 

A significant positive correlation was found between GEFC and REFC intensity and retinal thickness measured in the 1 central mm of the ETDRS grid (foveal thickness), with a Pearson’s correlation coefficient of *r* = 0.37 (*p* < 0.001) and *r* = 0.42 (*p* < 0.001) respectively. A significant positive correlation was also found between GEFC and retinal thickness measured in the inner nasal quadrant of the ETDRS grid: *r* = 0.49 (*p* < 0.001). No significant correlation was found between color-FAF parameters and retinal thickness in other quadrants of the ETDRS grid.

## 4. Discussion

In the present study we report on quantitative data obtained by means of color-FAF in patients affected by DM, with or without clinical signs of DR. Color-FAF was evaluated based on the stage of DR, systemic metabolic control, and either the presence or absence of DME. 

FAF, first introduced in clinical practice in the mid-1990s [24,25], has gained increasing importance in the diagnosis and follow-up of several retinal diseases. This non-invasive imaging technique allows for a metabolic mapping of endogenous fluorophores that physiologically or pathologically accumulate in the retina [10,24,25,26,27,28]. More recently, quantitative FAF (qAF), a new approach aimed to measure the intensity of short wavelength FAF by using confocal scanning laser ophthalmoloscopy (cSLO), has been developed [29,30] and applied to healthy eyes and to specific chorio-retinal diseases such as AMD and retinal dystrophies [31,32,33,34,35]. Most of the studies on FAF were performed using the HRA/Spectralis HRA (Heidelberg Engineering, Heidelberg, Germany) with excitation wavelength at 488 nm. However, a new cSLO system was recently introduced, the EIDON device, with a lower excitation wavelength (peak at 450 nm), which is thought to be able to excite a different range of fluorophores compared to 488 nm FAF. A specifically designed image analysis software allows to separately measure the contribution of the red-emitting (due in particular to lipofuscin) and green-emitting (due to coenzymes in redox reaction, AGEs, and collagen/elastin) spectral components [15,36]. This device has allowed a better characterization of the green-emitting fluorophores in healthy eyes, atrophic AMD, ABCA4-related retinopathy, and optic disc drusen [12,37,38,39,40]. However, to the best of our knowledge, no studies on qAF and quantitative color-FAF have been conducted on patients affected by DM so far.

Data on GEFC intensity in healthy subjects obtained in the present study are in agreement with a recent study by Borrelli et al. [37]. However, the absolute values cannot be directly compared between the two studies due to different characteristics of the populations included (such as higher mean age of the healthy population in the present study, with approximately 30 years of difference). Previous studies on qAF using 488nm-FAF and spectrofluorometric measurements (with excitation at 550 nm) have demonstrated that fundus autofluorescence in the macular region (excluding the fovea) progressively increased with age (starting from 20 years and increasing until 60 years) due to an increase in lipofuscin levels [41,42]. However, these studies did not register the modifications of FAF that were specifically determined by minor green-emitting fluorophores, but only focused on red-emitting lipofuscin. Therefore, further studies are needed to explore the topographical distribution of green-emitting fluorophores and their modifications with aging.

**Figure 2:**
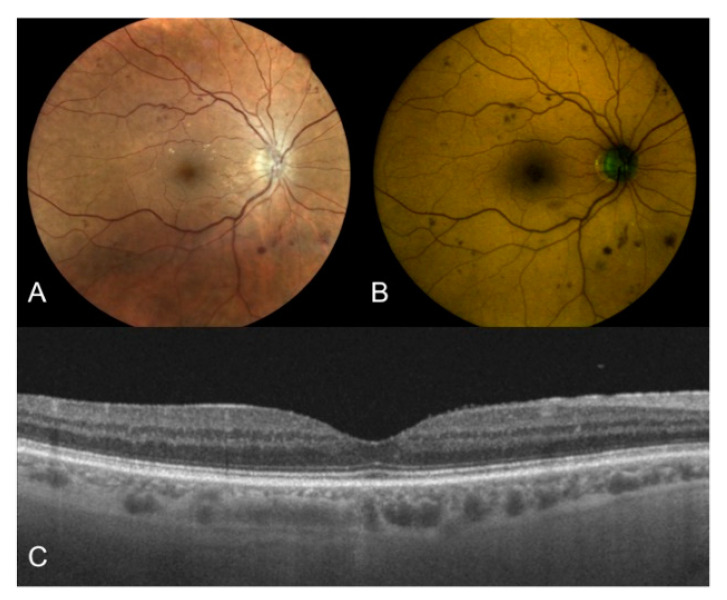
Right eye of a 68-year old male patient affected by type 2 DM with moderate DR. (**A**) True-color fundus photography of the posterior pole showing multiple hemorrhages and initial vitreo-retinal interface syndrome; (**B**) Color-FAF of the same eye with detected values of foveal GEFC and REFC intensity of 17 and 26, respectively; (**C**) OCT horizontal B-scan centred on the fovea showing a dry macula with normal reflectivity of the inner and outer retinal layers. DM: diabetes mellitus; DR: diabetic retinopathy; FAF: fundus autofluorescence; GEFC/REFC: green/red emission fluorescence components; OCT: optical coherence tomography.

In this study, patients with DM (with or without DR) presented higher values of both GEFC and REFC intensity in the foveal region compared to the control group, regardless of the presence of DME (Figure 2). As already mentioned, FAD, AGEs, and both collagen and elastin are minor fluorophores better excited by the shorter wavelength of this spectrally resolved FAF system. AGEs in particular are known to have a key role in the pathogenesis of DR [16]. AGEs are extracellular irreversible protein-bound compounds that progressively accumulate as a consequence of hyperglycemia [16,43]. Interestingly, however, we found no significant correlation between color-FAF parameters and the level of severity of DR, probably due to the fact that all included groups of patients had a good glycemic control (with no differences among the groups), as demonstrated by similar mean values of HbA1c. In a previous study by Hammer et al. with prototype color-FAF images obtained applying different filter combinations on 13 eyes with DR, the authors reported a green-shift in the appearance of FAF and speculated on its possible correlation with enhanced levels of AGEs in the retinas of patients with DM [43]. However, separate quantitative estimations of the contribution of the two emitting channels (red and green) were not reported [44].

**Figure 3:**
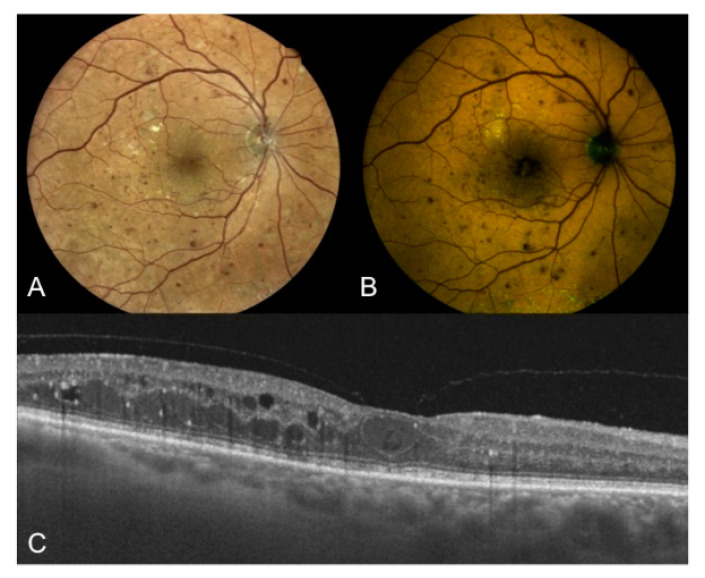
Right eye of a 67-year old male patient affected by type two DM with proliferative DR. (**A**) True-color fundus photography of the posterior pole showing NVD, multiple hemorrhages, extrafoveal hard exudates, cystoid macular edema, and peripheral laser treatment; (**B**) color-FAF of the same eye with detected values of foveal GEFC and REFC intensity of 31 and 40, respectively; (**C**) OCT horizontal B-scan centered on the fovea showing center involving cystoid macular edema (CRT = 356 μm), hard exudates, and hyper-reflective intraretinal spots. DM: diabetes mellitus; DR: diabetic retinopathy; NVD: new vessels at disc; FAF: fundus autofluorescence; GEFC/REFC: green/red emission fluorescence components; OCT: optical coherence tomography; CRT: central retinal thickness.

Based on data from the present study, higher levels of GEFC and REFC intensity were also present in eyes with center involving DME in the foveal area compared to eyes without this feature (Figure 3). Standard FAF was already used to characterize DME [7,8,9,45,46], and it showed 81% sensitivity and 69% specificity in detecting cystoid macular edema [47]. The origin of increased FAF in DME is still debated, however it was proposed that it could be due to accumulation of oxidative products as a consequence of microglia activation [7,11]. Additionally, as lipofuscin could be considered a marker of oxidative damage, and lipofuscin accumulates not only in the RPE but also in the microglia [7,11], we could hypothesize that both red-emitting (i.e., lipofuscin) and green-emitting (in particular AGEs) fluorophores are increased in the fovea of patients with DM and contribute to the final autofluorescence (as demonstrated by the increase in intensity of both components in this study). Another interesting finding of the present study supporting this hypothesis is that a positive correlation was found between GEFC and REFC intensity and foveal thickness, regardless of the presence of intraretinal cysts. Thus, it is unlikely that increased FAF could be simply and entirely due to luteal pigment displacement, thus allowing to better visualize fluorophores, as previously speculated [48]. Dong et al. demonstrated the existence of a positive correlation between retinal macular thickness and the aqueous levels of some pro-inflammatory cytokines (IL-1β, IL-6, IL-8, IP-10, MCP-1, and VEGF) in patients affected by type two DM, both with and without DME [49]. These pro-inflammatory molecules are produced by a variety of retinal cells [50], including activated microglia [51,52,53,54,55,56], an event occurring early in the pathogenesis of DR [51,52,53]. Therefore, our results may support the hypothesis that increased foveal FAF may represent an imaging biomarker of retinal inflammation in DM.

Lastly, contrary to what we observed in the foveal region, lower values of GEFC and REFC intensity were found in the outer superior and outer temporal macular quadrants of eyes with intraretinal fluid (in the corresponding quadrants) compared to eyes with dry retina in the same location. Studies conducted using qAF on healthy eyes detected the highest levels of FAF in the supero-temporal macular quadrants and the lowest levels in the infero-nasal quadrants; these studies correlated this finding with the spatial distribution of lipofuscin [41,42]. This result is controversial and difficult to interpret, and even if we could speculate on a different pattern of accumulation of fluorophores in retinas with DR or DME, studies on the topographical distribution of fluorophores (except lipofuscin in healthy eyes) are lacking and are needed in order to further understand the present data. 

Major limitations of the present study included the lack of longitudinal data in order to correlate data on color-FAF with the progression of DR/DME, and the low number of patients included in some groups of disease severity (e.g., mild DR and sight-threatening DR). Moreover, at the moment, data are limited to patients with pseudophakia or initial cataract, as 450nm-FAF is significantly influenced by media opacities, and only images with optimal quality should be analyzed in order to avoid gross errors. As already mentioned, another important limitation of the study is the fact that there’s a lack of histologic and immunohistochemical studies that could definitely confirm our hypothesis. Moreover, since the enrollment of patients was consecutive in a set period of time and not based on preliminary sample size calculations to reach a pre-determined statistical power in the comparisons among groups, we ended up with an unbalanced subdivision of patients among groups. In this case the smallest group determines the power of the comparisons. Therefore, some statistically significant differences among groups might not have been detected due to the limited power of the study. Therefore, this study should be considered as a pilot study to guide further investigations.

## 5. Conclusions

In conclusion, this is the first study to quantitatively evaluate data of color-FAF in patients with DM using a 450 nm FAF device. Higher levels of GEFC and REFC intensity were found in patients affected by DM compared to healthy, non-diabetic subjects, and in patients with DME compared to patients without DME. Moreover, a direct correlation between GEFC and REFC intensity and retinal thickness in the fovea was documented in patients with DM. We speculate that these results may be related to the accumulation, in particular, of AGEs and lipofuscin as the result of oxidative processes in response to the pro-inflammatory environment induced by hyperglycemia. Further histologic and immunohistochemical studies investigating accumulation of different fluorophores and their topographical distribution in the retina of patients affected by DM are needed to better understand the role of color-FAF in patients with DM and both DR and DME.

## Figures and Tables

**Table 1:** Main demographic and clinical data of the study population.

Group	Age (years)	BCVA (ETDRS Score)	DM Duration (years)	HbA1c (%)
Controls (18)	54.9 ± 15.7	85 ± 0.0 ^†^		
No DR (39)	63.1 ± 16.7	83.8 ± 2.2 ^‡^	9.5 ± 7.1	6.7 ± 1.3
Mild DR (22)	63.1 ± 19.6	82.3 ± 4.9 ^†^	18 ± 9.6 ^¶^	7.2 ± 0.9
Moderate DR (100)	69.41 ± 12.3 ^§^	75.4 ± 11.4	15.2 ± 9.2 ^¶^	7.6 ± 1.3
Sight-threatening DR (32)	60.4 ± 15.7	76.3 ± 9.8	16.6 ± 8.5 ^¶^	7.4 ± 1.5
*p* value *	0.013	<0.001	<0.001	0.21

* One-way ANOVA analyses: comparison among controls, patients with DM and no DR, and patients with DM and different stages of DR severity. Comparison versus controls: ^§^ Scheffé’s test, *p* = 0.03. Comparison versus DM no DR: ^¶^ Scheffé’s test, *p* < 0.01. Comparison versus moderate DR: ^†^ Scheffé’s test, *p* = 0.04; ^‡^ Scheffé’s test, *p* < 0.01. Statistical significance was set at *p* = 0.05. Values are reported as mean ± SD. BCVA: best-corrected visual acuity; HbA1c: glycated hemoglobin; DM: diabetes mellitus; DR: diabetic retinopathy.

**Table 2:** Comparison between GEFC and REFC intensity values in eyes with and without diabetic macular edema.

Macular Sector (ETDRS Grid)	DME (no = 0/yes = 1)	GEFC Intensity	*p* Value *	REFC Intensity	*p* Value *
Fovea	0 1	17.4 ± 14.1 43.3 ± 21.2	<0.001 **	18.5 ± 12.6 47.1 ± 21.4	<0.001 **
Nasal 1.5 mm	0 1	52.2 ± 25.9 54.7 ± 21.2	0.66	62 ± 23.3 62.7 ± 21.1	0.90
Nasal 3 mm	0 1	74.9 ± 23.5 68.5 ± 15.4	0.35	96.4 ± 25.3 88.6 ± 17.3	0.30
Superior 1.5 mm	0 1	53.2 ± 19.3 52 ± 21.6	0.38	64.2 ± 19.7 61 ± 21.7	0.44
Superior 3 mm	0 1	87.9 ± 21.9 75.2 ± 25.3	0.03 **	111.7 ± 26.7 89.2 ± 23.7	0.001 **
Inferior 1.5 mm	0 1	55.2 ± 18.4 53.7 ± 15.2	0.66	73.5 ± 79.8 62.5 ± 16.5	0.42
Inferior 3 mm	0 1	78.5 ± 20.6 74.7 ± 20.7	0.43	104.4 ± 88.9 93.2 ± 6.1	0.58
Temporal 1.5 mm	0 1	55.9 ± 19.7 50.7 ± 19.4	0.11	65.9 ± 19.8 60.3 ± 19.4	0.10
Temporal 3 mm	0 1	78.4 ± 21.8 64.9 ± 23.6	0.004 **	95 ± 23.1 79.4 ± 26.3	0.002 **

* Two-sided unpaired t-test: comparison between patients with and without DME in nine different sectors of a standard 6-mm ETDRS grid. Statistical significance was set at *p* = 0.05. ** Comparisons that reached statistical significance. Values are reported as mean ± SD. DME: diabetic macular edema; GEFC: green emission fluorescence component; REFC: red emission fluorescence component; SD: standard deviation; ETDRS: early treatment diabetic retinopathy study.

## Data Availability

Data are available upon request to the corresponding author.
